# Propolis as a Treatment Option for Hand, Foot, and Mouth Disease (HFMD) in Children: A Prospective Randomized Clinical Study

**DOI:** 10.3390/children12060695

**Published:** 2025-05-29

**Authors:** Manolya Kara, Murat Sütçü, Ömer Kılıç, Doruk Gül, Tugçe Tural Kara, Gulşen Akkoç, Ayşe Baktır, Şefika Elmas Bozdemir, Özlem Özgür Gündeşlioğlu, Funda Yıldız, Ciğdem Yanar Ayanoğlu, Meltem Bozacı Kılıçoğlu, Raif Yıldız, Ateş Kara

**Affiliations:** 1 Department of Pediatric Infectious Diseases, Faculty of Medicine, Yeditepe University, 34755 Istanbul, Turkey; 2 Department of Pediatric Infectious Diseases, Faculty of Medicine, İstinye University, 34010 Istanbul, Turkey; murat.sutcu@istinye.edu.tr; 3 Department of Pediatric Infectious Diseases, Faculty of Medicine, Eskisehir Osmangazi University, 26040 Eskisehir, Turkey; okilic@ogu.edu.tr; 4 Department of Pediatrics, Faculty of Medicine, Istinye University, 34010 Istanbul, Turkey; doruk.gul@istinye.edu.tr (D.G.); funda.yildiz@istinye.edu.tr (F.Y.); 5 Department of Pediatric Infectious Diseases, Faculty of Medicine, Akdeniz University, 07070 Antalya, Turkey; tugcekara@akdeniz.edu.tr; 6 Department of Pediatric Infectious Diseases, Van Education and Research Hospital, 65300 Van, Turkey; gulsen.akkoc@marmara.edu.tr; 7 Department of Pediatric Infectious Diseases, Faculty of Medicine, Istanbul University, 34093 Istanbul, Turkey; ayse.baktir@istanbul.edu.tr; 8 Department of Pediatric Infectious Diseases, Dörtçelik Children’s Hospital, 16120 Bursa, Turkey; dortcelikchh@saglik.gov.tr; 9 Department of Pediatric Infectious Diseases, Faculty of Medicine, Cukurova University, 01330 Adana, Turkey; oozgur@cu.edu.tr; 10 Department of Pediatrics, Faculty of Medicine, Yeditepe University, 34755 Istanbul, Turkey; cigdem.ayanoglu@yeditepe.edu.tr; 11 Department of Otolaryngology, Faculty of Medicine, Yeditepe University, 34755 Istanbul, Turkey; meltem.kilicoglu@yeditepe.edu.tr; 12 Department of Emergency Medicine, Basaksehir Cam Sakura Training and Research Hospital, University of Health Sciences, 34480 Istanbul, Turkey; raif.yildiz@istanbul.edu.tr; 13 Department of Pediatric Infectious Diseases, Faculty of Medicine, Hacettepe University, 06080 Ankara, Turkey; ates.kara@hacettepe.edu.tr

**Keywords:** hand, foot, and mouth disease (HFMD), propolis, enteroviruses

## Abstract

**Background**: Hand, foot, and mouth disease (HFMD) is a frequently self-limited viral infectious disease that affects children and has no specific antiviral treatment option. There has been increasing interest in bee products in recent years, and propolis has come to the fore due to its impressive therapeutic and protective effects. Although previous studies have shown the inhibitory effect of propolis against enteroviruses (EVs), there are no clinical data regarding its use in combatting HFMD. This prospective multicenter randomized clinical study aimed to evaluate the effect of administering propolis to children with HFMD. **Methods**: This study analyzed 183 children with HFMD. All children were assessed for eligibility and diagnosed with HFMD by a child health and disease specialist after presenting with symptoms of either fever, enanthem, or exanthems that had begun within the last 48 h. The patients were randomly assigned to the group receiving Anatolian propolis (n = 87) or that receiving no supplement—the control group (n = 96)—in addition to receiving symptomatic therapy as decided by the physician. The duration of the patient’s complaints, the distribution of the lesions on their body, and their fever status were recorded on admission. Parents were asked to rate the severity of their child’s restlessness, inappetence, and sleeplessness on a scale of 0–10 at their initial, second (at 48th hour), and third (after 5–7 days) visits to the hospital. The primary data analysis methods included the Kolmogorov–Smirnov test for normality and non-parametric tests such as the Kruskal–Wallis and Mann–Whitney U tests, which were used for group comparisons. **Results**: The median age of the patients was 28 months (range: 12–112), and the male-to-female ratio was 1:1. Most patients (62.8%) had no history of a household contact with HFMD. Intraoral lesions were present in 92.3% of patients, and 47.5% received the propolis treatment while 53.5% were in the control group. There was a significant difference between the groups in terms of their complaint scores during their second (*p* = 0.028) and third (*p* < 0.001) visits to the hospital. In addition, the mean duration of the illness in the propolis group (7.45 days) was significantly lower than that in the control group (8.58 days) (*p* < 0.001). No adverse effects were observed. **Conclusions**: Propolis has been shown to facilitate symptomatic relief and reduce the duration of the disease in children with HFMD. To better assess the efficacy of this product, which can be used safely in children, future studies supported by virological analyses are required.

## 1. Background

Hand, foot, and mouth disease (HFMD) is one of the most well-known viral infections found in children and adults, and is caused primarily by Coxsackievirus A16 (CV-A16) and Enterovirus A71 (EV-71) [1]. The disease is characterized by oral enanthem and often non-pruritic exanthems that involve the hands, feet, buttocks, legs, and arms [2]. Although the disease is often self-limiting, in some cases, it may progress with complications such as aseptic meningitis, acute cerebellar ataxia, polio-like syndrome, encephalitis, and Guillain–Barré syndrome [3]. Clinical cases associated with EV-71 have been observed to be more severe, and the virus is believed to trigger gray matter damage, resulting in motor dysfunction [4]. In rare cases, interstitial pneumonia, myocarditis, pancreatitis, and pulmonary edema may be observed during the course of the disease [5].

Even when it does not cause serious complications, HFMD causes a variety of socioeconomic issues due to it being highly contagious in the environment; it can cause family and school epidemics as well as school and workforce closures. There is no specific treatment for HFMD. Adequate hydration, fever control with acetaminophen and ibuprofen, and antihistamine agents can be used for symptomatic treatment [3]. Antiviral medicine and vaccine research are ongoing, particularly against EV-caused HFMD [6].

Propolis is a complex resinous mixture produced by forager honeybees (*Apis mellifera*) that is made from plant exudates and resins (e.g., buds and twigs) blended with beeswax and salivary enzymes [7]. It has been used for centuries due to its antioxidant, anti-microbial, and anti-inflammatory properties [8]. Its antiviral activity against several respiratory (adenovirus, coronaviruses, influenza virus, rhinovirus, etc.) and non-respiratory viruses (herpes simplex virus type 1 and 2, human immunodeficiency virus, enterovirus, and poliovirus type 1) has also been demonstrated [8,9,10]. Furthermore, randomized trials have shown that propolis, when used as treatment for upper respiratory tract infections, including Severe Acute Respiratory Syndrome Coronavirus 2 (SARS-CoV-2), improves clinical outcomes and shortens the duration of symptoms without leading to serious side effects [7,11].

To date, no studies have been conducted on the use of propolis in the treatment of hand, foot, and mouth disease (HFMD), despite its known antiviral activity against enteroviruses (EVs). Thus, the present study aimed to assess the efficacy of propolis in alleviating the clinical symptoms of children diagnosed with HFMD.

## 2. Methods

This multicenter randomized controlled study was conducted from January 2019 to January 2022 across 8 centers in Turkey. The protocol was approved by the Eskisehir Osmangazi University Interventional Research Ethics Committee (date: 25 April 2019, number: 80558721-050.99-E.49493) and conducted under the principles of Good Clinical Practice. This study is registered at ClinicalTrials.gov (NCT06455007). Written informed consent was obtained from the parents of all patients included in the study.

Initially, 220 patients who were examined by a child health and disease specialist and diagnosed with HFMD (with symptoms of either a fever, enanthem, or exanthems beginning within the last 48 h) were assessed for eligibility. The patients whose complaints had been ongoing for longer than 48 h, those whose parents stated that they could not comply with the follow-ups, those who were taking another antiviral or supportive treatment, those who had used antibiotics in the last month, those with a history of immunodeficiency or a family history of immunodeficiency, those who had a history of anaphylaxis due to any support product or drug, and patients with a chronic disease or skin lesions were excluded from the study.

Upon their first admission, the duration of the patient’s complaints, the distribution of the lesions on their body, and their fever status were recorded. Parents were asked to rate the severity of their child’s restlessness, inappetence, and sleeplessness on a scale of 0–10.

A local member of the research team used a centralized computerized randomization method (RAND2 randomization software [version 2.1], The MathWorks Inc., Natick, MA, USA, contractually maintained by the data management team) to assign participants 1:1 to one of the two trial arms. To guarantee a uniform distribution of the groups across all research sites, lists were added to the automatic online randomization method in four blocks.

The sample size was calculated based on data from similar studies. Previous research has reported a mean difference of 1.5 points and a standard deviation of 2.5 in children’s restlessness scores between treatment and control groups. With a power of 90% and a significance level (alpha) of 5%, at least 80 participants in each group were required. Patients with chronic illnesses and cases that were not admitted to the hospital within 72 h of symptom onset were excluded from this study.

All patients were followed up with twice more, 48 h after their first admission (2nd visit) and on the 5th–7th day after their admission (3rd visit). Another phone evaluation was conducted for those with continued complaints after the last follow-up. During these visits, the parents were asked to rate their child’s fever status, restlessness, inappetence, and sleeplessness again, and these values were recorded. Patients’ adherence to the medication and side effects from the drug were evaluated. After the patient’s recovery, the disease’s total duration and all symptoms were recorded. Patients who were hospitalized or developed complications were noted.

### Propolis Preparation

BEE’O UP (BEE&YOU) Propolis Drops contain Anatolian propolis extract the innovation-award winner and patented extraction technology developed in ARI Teknokent laboratories of Istanbul Technical University. Anatolian Propolis Extract (A.P.E®) is obtained from the pristine Anatolian mountains, one of the world’s most biodiverse regions, and is produced as a standardized with patented technology. Based on the spectrophotometric analysis report published for this product, its minimum phenolic content per ml is 106.0 mg GAE, its flavonoid content is 73.1 mg ke (kaempferol equivalent), and its total content is 253.9 mg te (Trolox Equivalent)/mL (Table 1).

The total phenolic content of BEE’O UP (BEE&YOU) Propolis Drops (30%) is based on the results of an analysis performed on 10 June.2020 in the R&D Laboratory of SBS Scientific Bio Solutions and the Chemistry Department Laboratory of Science Faculty of Karadeniz Technical University. The phenolic and flavonoid component content of BEE’O UP (BEE&YOU) Propolis Drops (30%) is as listed in the table below.

Patients in both groups were prescribed antipyretics and antihistamines when deemed appropriate by the clinician. Propolis drops were administered orally, three times a day for 7 days, to patients in the propolis group, with 10 drops constituting a dose. The drops were placed directly in the mouth using a dropper. The same batch of propolis drops was used for all patients. Both the investigators and the patients were aware of the drug being given.

Analyses were performed using a prospectively defined analysis plan. Using SPSS v28.0 (Statistical Product and Service Solutions, IBM Corp., Armonk, NY, USA), categorical variables were presented as numbers (n) and percentages (%). The assumption of normality was assessed using the Kolmogorov–Smirnov test. Since the data were not normally distributed, non-parametric tests (Kruskal–Wallis and Mann–Whitney U test) and parametric tests (Student’s *t*-test) were used for group comparisons. Descriptive analyses were presented using medians and the interquartile range (IQR). A *p*-value of less than 0.05 was considered to indicate a statistically significant result.

## 3. Results

**Figure 1:**
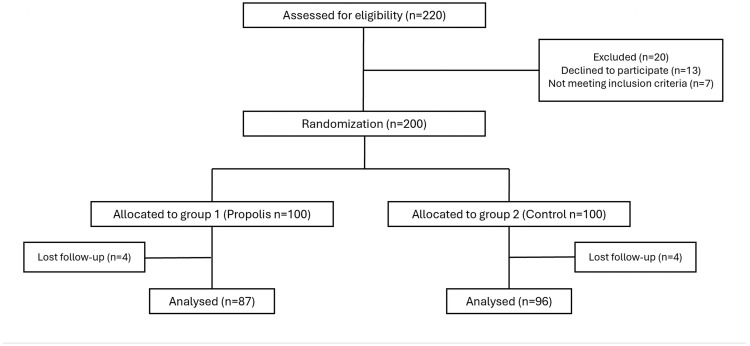
Patient enrollment process.

Two hundred patients were randomly assigned to the two groups (however, thirteen declined to participate and seven did not meet the inclusion criteria). A total of 17 patients were lost to follow-up, resulting in 183 patients’ data [propolis group (n = 87) and control group (n = 96)] finally being analyzed (Figure 1).

The patients’ median age was 28 (12–112) months, and the male–female ratio was 1.1. Additionally, 115 (62.8%) of the patients had no history of household contact (Table 2).

The number of patients with a history of sibling contact was 25 (13.7%), while 28 (15.3%) had contact with cousins. Furthermore, 15 (8.2%) patients had contact with other people with HFMD. At the time of their admission to the hospital, 117 (63.9%) patients first experienced symptoms the day before, while 66 (36.1%) patients had been experiencing symptoms for 2 days.

A total of 169 (92.3%) patients had intraoral lesions. Of these, 55 (30.1%) patients had lip lesions and 65 (35.5%) had tongue lesions. The number of patients with lesions on their palate was 159 (86.9%). In addition, 51 (27.9%) patients had lesions on their cheeks, while 68 (37.1%) patients had rashes only on their extremities, and 75 (41%) had rashes on their trunk and extremities. Additionally, 40 (21.9%) patients had a rash all over their whole body.

Subsequently, 87 (47.5%) patients were prescribed propolis, while 96 (53.5%) formed the control group. While 117 (63.9%) patients were not prescribed any other medication, 48 (26.2%) were prescribed paracetamol and 18 (9.8%) were prescribed antihistamines.

The median age of the group prescribed propolis was 28 (12–112) months and the median age of the control group was 29.5 (12–112) months. There was no statistically significant difference between the ages of the two groups (*p* = 0.967).

At the beginning of the treatment, 19 (21.8%) of the patients given propolis had a body temperature below 37.5 °C, 40 (46%) had a temperature between 37.5 and 38.5 °C and 28 (32.2%) had one over 38.5 °C. In the control group, 35 (36.5%) had a body temperature below 37.5 °C, 40 (41.7%) had a temperature between 37.5 °C and 38.5 °C, and 21 (21.9%) had one over 38.5 °C.

**Figure 2:**
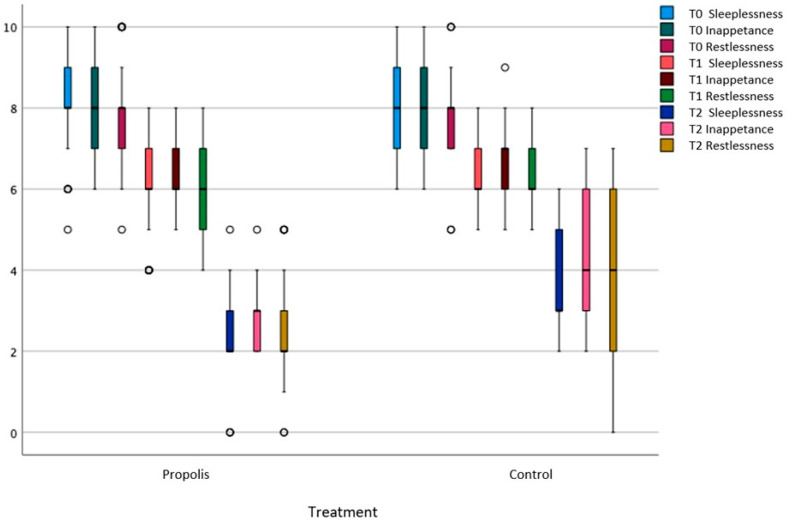
Complaint scores at 2nd and 3rd visits. Complaint scores for sleeplessness, inappetence, and restlessness at the baseline (T0), second visit (T1), and third visit (T2) are shown for both the propolis and control groups. In the propolis group, all complaint scores decreased significantly over time, while the control group showed only slight reductions. These findings indicate that propolis may be more effective than the control in reducing these symptoms.

There was no significant difference between the propolis and control groups in terms of restlessness, inappetence, and sleeplessness at the time of their admission to the hospital. However, a significant difference was found between the groups in terms of their complaint scores at their second (*p* = 0.028) and third visits (*p* < 0.001) (Table 3 and Figure 2).

**Figure 3:**
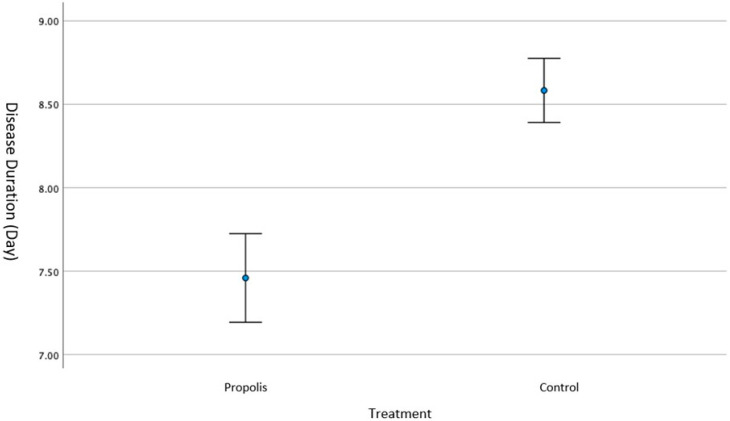
Comparison of disease duration (days) between the propolis and control groups. The mean disease duration was significantly shorter in the propolis group compared to the control group (Student’s *t*-test, *p* < 0.001).

The mean ± SD disease duration for propolis recipients was 7.45 ± 0.80 days (95 Cl%: 5.82–6.31), and the mean ± SD disease duration for the control group was 8.58 ± 0.94 days (95 Cl%: 8.39–8.77). A statistically significant difference was found between the treatment and control groups in terms of the duration of the disease (*p* < 0.001) (Figure 3).

No side effects were observed, except for a dislike of the taste, which was reported in eight patients in the propolis group.

## 4. Discussion

Patients and physicians have begun to favor herbal medicine for widespread viral infections that impact numerous individuals and have no specific treatment. Propolis, a resinous honeybee product, has lately gained popularity in such cases [7]. It is a mixture of resin, wax, essential oil, pollen, other minerals, vitamins, poly and oligosaccharides, and phenolic compounds (e.g., flavonoids, aromatic acids, and esters) [7].

Anatolian geography has created a rich and extremely diverse endemic flora [12]. Bee products obtained from this region are thus also high-quality and have nutritional benefits. The results of an analysis of its antioxidant capacity have shown that Anatolian propolis contains 15 different phenolic and flavonoid components, making it rich in polyphenols. These components are caffeic acid phenethyl ester (CAPE), caffeic acid, quercetin, galangin, chlorogenic acid, linolenic acid, palmitic acid, hydroxytyrosol, coumaric acid, apigenin, chrysin, and pinocembrin. The total antioxidant capacity of Anatolian propolis was also found to be high [12,13].

Propolis has been used to heal or mitigate the effects of various diseases since ancient times [14]. The Egyptians utilized propolis to treat diseases and embalm the dead, and the Greeks and Romans employed it as a medicine to treat skin abscesses [15]. Recent studies have revealed that it has many anti-infective, antioxidant, immune-regulatory, and antiproliferative functions [15,16,17,18]. Propolis has been shown, in vitro and in vivo, to have broad-spectrum effects on viral infectivity and replication, as well as modulatory effects on cytokine production and immune cell activation, both of which are part of both innate and adaptive immune responses [19].

Yıldırım et al. [9] analyzed the antiviral activity of propolis against HSV-1 and HSV-2 viruses and demonstrated that viral replication was significantly suppressed in the presence of propolis. Furthermore, the combination of propolis and acyclovir led to a synergistic effect, outperforming acyclovir alone [9]. Propolis has also demonstrated antiviral activity against several respiratory and non-respiratory viruses [8,9,10]. Szmeja et al. [20] examined the clinical impact of propolis on rhinovirus infection. They found that in the propolis-treated group, the duration of illness was shortened by 2.5-fold, with symptoms subsiding on the first day of treatment and full recovery achieved within three days.

Despite often having a mild and self-limiting disease course, HFMD can, in rare cases, lead to serious complications such as myocarditis, meningoencephalitis, and flaccid paralysis [2]. Particularly at young ages, high and prolonged fever and clinical symptoms including lethargy, vomiting, and the presence of EV-71 are related to severe HFMD [21]. In uncomplicated cases, full recovery takes 7–10 days [2]. However, depending on the type of EV and the severity of the illness, the virus can continue to be excreted in the feces for up to 10 weeks and can linger in the respiratory tract for about 30 days [22,23,24]. During this period, outbreaks can occur within the patients’ households, daycare centers, and schools. Management is mainly supportive as no specific antiviral therapy has been approved for the treatment of EVs.

Propolis was recently shown to have antiviral activity and to reduce the replication of EV surrogates in a time- and concentration-dependent manner in an in vitro study [10]. To our knowledge, there are no clinical studies on propolis and EV in the literature. In the current study of children with HFMD, a significant difference in complaint scores was found between the propolis and control groups on the 2nd-day and 5–7th-day follow-ups. In the propolis group, the scores for restlessness, inappetence, and sleeplessness were significantly lower. In addition, the mean disease duration was shorter in the propolis group. This may be related to a lower viral load due to decreased viral replication and the anti-inflammatory properties of propolis. While there is no specific antiviral treatment for HFMD, early improvements in symptoms may lead to less symptomatic drug use, increased oral nutrition, and fewer hospitalizations. In the aforementioned study by Silva-Beltrán et al. [10], a reduction in enteric viral replication was also demonstrated. The effect of propolis on enteric virus shedding can reduce a virus’s transmission rate and the incidence of outbreaks.

The preventive effects of propolis against several diseases, including COVID-19 infection, have also been shown in various studies [25,26]. However, in their clinical studies, Antunes Moura et al. [27] found that the use of propolis did not decrease the frequency of infections in children over 1 year old. The authors concluded that this result was due to an insufficient dilution (3%) for effective action. It is unknown whether it had an effect on EVs and whether the use of a prophylactic after exposure would prevent disease development. The effects of propolis on the shedding of various viruses and its ability to protect against these diseases are areas for future study. 

There have been no reports of significant side effects from propolis, which is a substance that has been utilized since ancient times [28]. Likewise, no propolis-related side effects were observed in the present research. However, it is a known fact that the content of propolis can vary significantly depending on where it is produced [13]. While this type of herbal medicine is recommended, especially for children, its purity and contents should be well known before its use.

### Strengths and Limitations of This Study

The present study has several limitations. Firstly, although patient randomization was performed blindly using a computerized system, the propolis supplementation phase was conducted as an open-label intervention since both the attending physician and the patient’s families knew about the treatment. The absence of a placebo-controlled group is a notable limitation of this study. However, using objective evaluation criteria during patient assessments likely reduced the risk of bias associated with self-reported outcomes.

The second limitation of this study is its reliance on clinical findings and the lack of virological assessments. However, laboratory tests are not commonly used in many centers to diagnose HFMD. A diagnosis is typically made based on the physician’s clinical evaluation of the case. The fact that each physician involved in the study was a pediatric specialist eliminates any potential doubts regarding the diagnosis. Nevertheless, future virological-based studies may provide more subjective data regarding the change in viral load before and after treatment.

Despite these limitations, to the best of our knowledge, this study is the first to evaluate the use of propolis in patients diagnosed with HFMD, a condition for which no antiviral treatment options are currently available, and to report symptomatic improvement. Our study is relevant as it could guide future research.

## 5. Conclusions

In conclusion, the use of propolis in children with HFMD has been shown to facilitate symptomatic relief and reduce the duration of the disease. To better assess the efficacy of this product, which can be used safely in children, future studies supported by virological analyses are required.

## Figures and Tables

**Table 1:** Results of analysis of BEE’O UP (BEE&YOU) Propolis Drops (30%).

Content Information Declared on the Label	Pure Anatolian Propolis (30%), water–ethanol solution
pH	5.10 ± 0.02
Total polyphenol content (mg GA/mL)	59.3 ± 0.93
Total flavonoid content (mg GA/mL)	22.3 ± 0.23
Total antioxidant capacity (FRAP mmol FeSO_4_⋅7H_2_O/mL)	858.9 ± 0.74
Results of Phenolic Component Analysis	Results (mcg/mL sample)
p-Hydroxybenzoic acid	143.0
Epicatechin	312.0
Caffeic acid	1520.0
p-Coumaric acid	406.8
Ferulic acid	977.2
Resveratrol	45.3
Luteolin	38.7
Quercetin	186.3
t-Cinnamic acid	158.8
Apigenin	451.2
Hesperetin	384.8
Rhamnetin	610.0
Chrysin	5942.0
Pinocembrin	1036.2
Caffeic acid phenethyl ester (CAPE)	16,792.0

GA: gallic acid; FRAP: ferric reducing antioxidant power.

**Table 2:** Baseline demographic characteristics of patients.

Parameter (*N*)	All Patients (183)	Propolis (87)	Control (96)
Age (month, median, range)	28 (12–112)	26 (12–110)	28 (12–112)
Sex, female (*n*, %)	87 (47.5)	42 (48.3)	45 (46.9)
Pre-admission complaint time			
1 day ago	117 (63.9)	56 (64.4)	61 (63.5)
2 days ago	66 (36.1)	31 (35.6)	35 (36.5)
Household contact *n* (%)	68 (37.2%)	32 (36.8)	36 (37.5)
Distribution of intraoral lesions (*n*, %)			
Lips	55 (30.1)	26 (29.9)	29 (30.2)
Tongue	65 (35.5)	31 (35.6)	34 (35.4)
Cheeks	51 (27.9)	24 (27.6)	27 (28.1)
Palatine	159 (86.9)	78 (89.7)	81 (84.4)
Distribution of rashes (*n*, %)			
Extremities only	68 (37.1)	33 (37.9)	35 (36.5)
Trunk and extremities	75 (41)	36 (41.4)	39 (40.6)
Whole body	40 (21.9)	18 (20.7)	22 (22.9)
Presence of fever (*n*, %)			
<37.5	(25.5)	19 (21.8)	35 (36.5)
37.5–38.5	101 (48.6)	40 (46)	40 (41.7)
≥38.5	54 (25.9)	28 (32.2)	21 (21.9)

**Table 3:** Median (25–75%) complaint scores of propolis and control groups as measured before treatment, after 48 h of treatment, and after 5–7 days of treatment.

	Propolis Median (25–75%)	Control Median (25–75%)	*p* Value
Before treatment			
Restlessness	8 (8–9)	8 (7–9)	0.824
Inappetence	8 (7–9)	8 (7–9)	0.726
Sleeplessness	8 (7–8)	8 (7–8)	0.787
After 48 h of treatment			
Restlessness	6 (6–7)	6 (6–7)	0.569 *
Inappetence	6 (6–7)	7 (6–7)	**0.056**
Sleeplessness	6 (5–7)	6 (6–7)	**0.028**
After days 5–7 of treatment			
Restlessness	2 (2–3)	3 (3–5)	**<0.001**
Inappetence	3 (2–3)	4 (3–6)	**<0.001**
Sleeplessness	2 (2–3)	4 (2–6)	**<0.001**

* *p* < 0.05 is statistically significant.

## Data Availability

The original contributions presented in this study are included in the article. Further inquiries can be directed to the corresponding author.
